# Crystal structure of *rac*-(3a*R*,4*S*,5a*R*,6*S*,9*R*,10a*S*,10b*R*)-3a,5a,9-tri­methyl­tetra­deca­hydro-6,9-ep­oxy­cyclo­hepta­[*e*]inden-4-ol monohydrate

**DOI:** 10.1107/S2056989015015698

**Published:** 2015-08-29

**Authors:** Andreas Schäfer, Christopher Golz, Hans Preut, Carsten Strohmann, Martin Hiersemann

**Affiliations:** a Fakultät Chemie und Chemische Biologie, Technische Universität Dortmund, Otto-Hahn-Strasse 6, 44221 Dortmund, Germany

**Keywords:** crystal structure, hydrate, hydrogen bonding, diterpenoid synthesis

## Abstract

The title hydrate, C_17_H_28_O_2_·H_2_O, was synthesized in order to determine the relative configuration of the tetra­cyclic framework. The fused 5,6,7-tricarbocyclic core exhibits an entire *cis*-annulation, featuring a 1,4-*cis*-relation of the angular methyl groups in the six-membered ring. The oxa bridge of the ep­oxy­cyclo­heptane moiety is oriented towards the concave face of the boat-shaped mol­ecule, whereas the angular methyl groups are directed towards the convex face. The asymmetric unit of the crystal contains two nearly identical formula units, which are related *via* a pseudo-centre of symmetry. The structure could be solved in the space groups *I*-4 and *I*4_1_/*a*. The refinement in the acentric space group, however, gave significantly better results and these are used in this paper. O—H⋯O hydrogen bonds are observed between the organic mol­ecules, between the organic mol­ecules and the water mol­ecules, and between the water mol­ecules, forming a chain along the *c-*axis direction.

## Related literature   

For the total synthesis of jatrophane diterpenoids, see: Schnabel & amp; Hiersemann (2009 ▸); Schnabel *et al.* (2011 ▸). For the synthesis of norjatrophane diterpenoids, see: Helmboldt *et al.* (2006 ▸); Helmboldt & Hiersemann (2009 ▸).
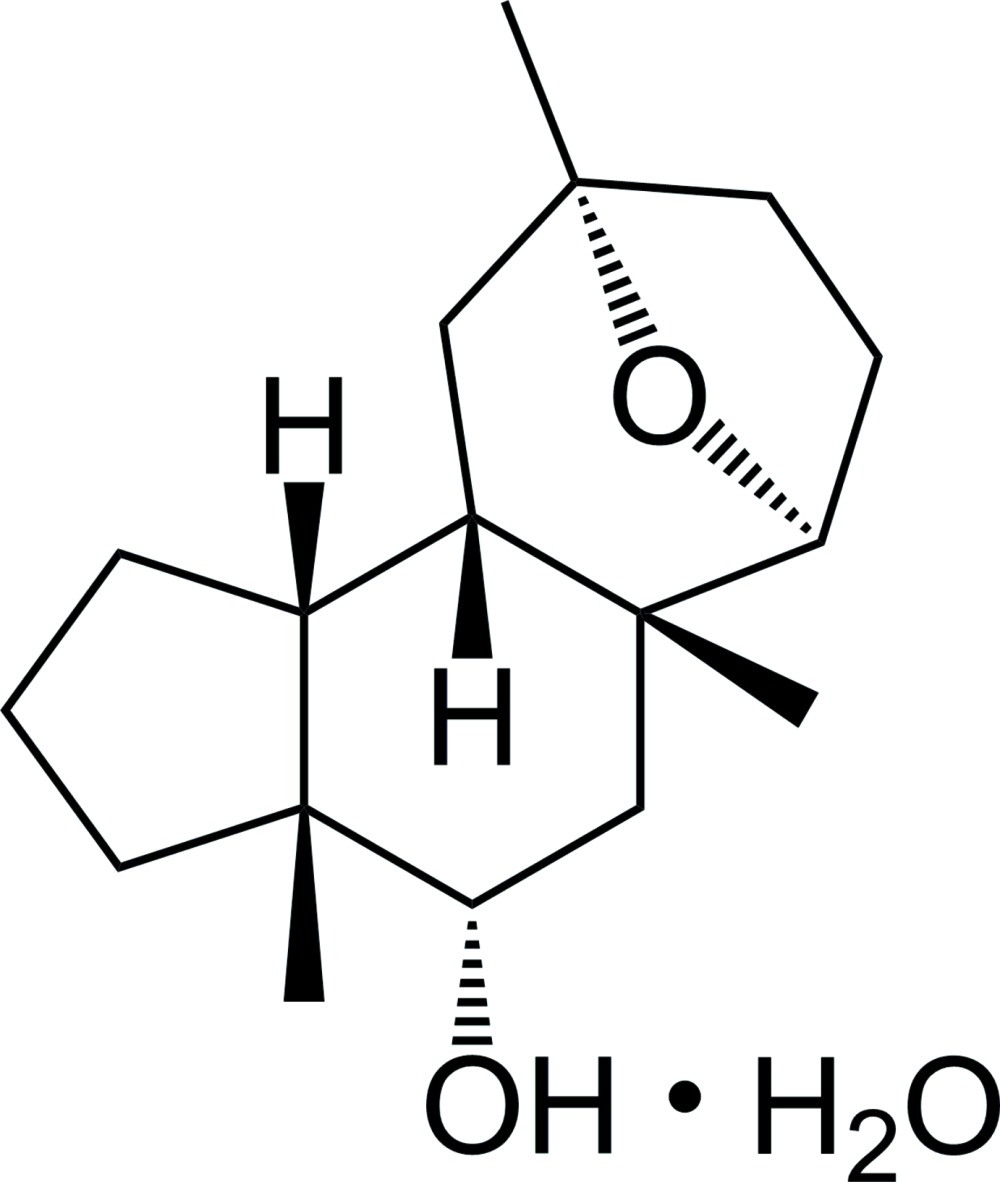


## Experimental   

### Crystal data  

C_17_H_28_O_2_·H_2_O
*M* *_r_* = 282.41
Tetragonal, 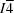

*a* = 25.3756 (9) Å
*c* = 9.6397 (6) Å
*V* = 6207.2 (6) Å^3^
*Z* = 16
Mo *K*α radiation
μ = 0.08 mm^−1^
*T* = 173 K
0.32 × 0.13 × 0.10 mm

### Data collection  

Agilent Xcalibur Sapphire3 diffractometer
Absorption correction: multi-scan (*CrysAlis PRO*; Agilent, 2014) *T*_min_ = 0.978, *T*_max_ = 1.0
36469 measured reflections
6781 independent reflections
4846 reflections with *I* > 2σ(*I*)
*R*_int_ = 0.087

### Refinement  

*R*[*F* ^2^ > 2σ(*F* ^2^)] = 0.051
*wR*(*F* ^2^) = 0.109
*S* = 1.03
6781 reflections
391 parameters
6 restraints
H atoms treated by a mixture of independent and constrained refinement
Δρ_max_ = 0.19 e Å^−3^
Δρ_min_ = −0.27 e Å^−3^

### 

Data collection: *CrysAlis CCD* (Oxford Diffraction, 2008 ▸); cell refinement: *CrysAlis CCD*; data reduction: *CrysAlis CCD*; program(s) used to solve structure: *SHELXD* (Sheldrick, 2008 ▸); program(s) used to refine structure: *SHELXL2013* (Sheldrick, 2015 ▸); molecular graphics: *SHELXTL-Plus* (Sheldrick, 2008 ▸); software used to prepare material for publication: *SHELXL97* (Sheldrick, 2008 ▸) and *PLATON* (Spek, 2009 ▸).

## Supplementary Material

Crystal structure: contains datablock(s) I, 3000. DOI: 10.1107/S2056989015015698/fk2090sup1.cif

Structure factors: contains datablock(s) I. DOI: 10.1107/S2056989015015698/fk2090Isup2.hkl

Supporting information file. DOI: 10.1107/S2056989015015698/fk2090Isup3.cml

**Fig. 1.**
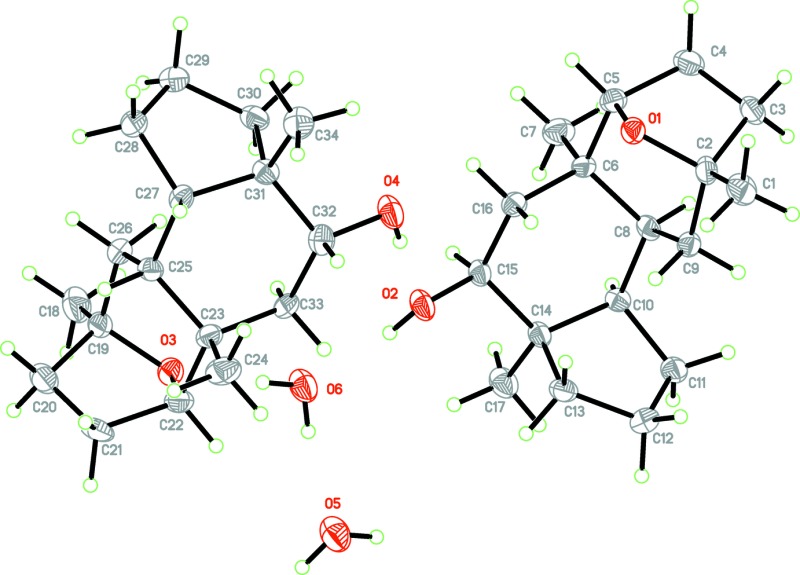
: The molecular structure of the title compound with anisotropic displacement ellipsoids drawn at the 50% probability level.

**Fig. 2.**
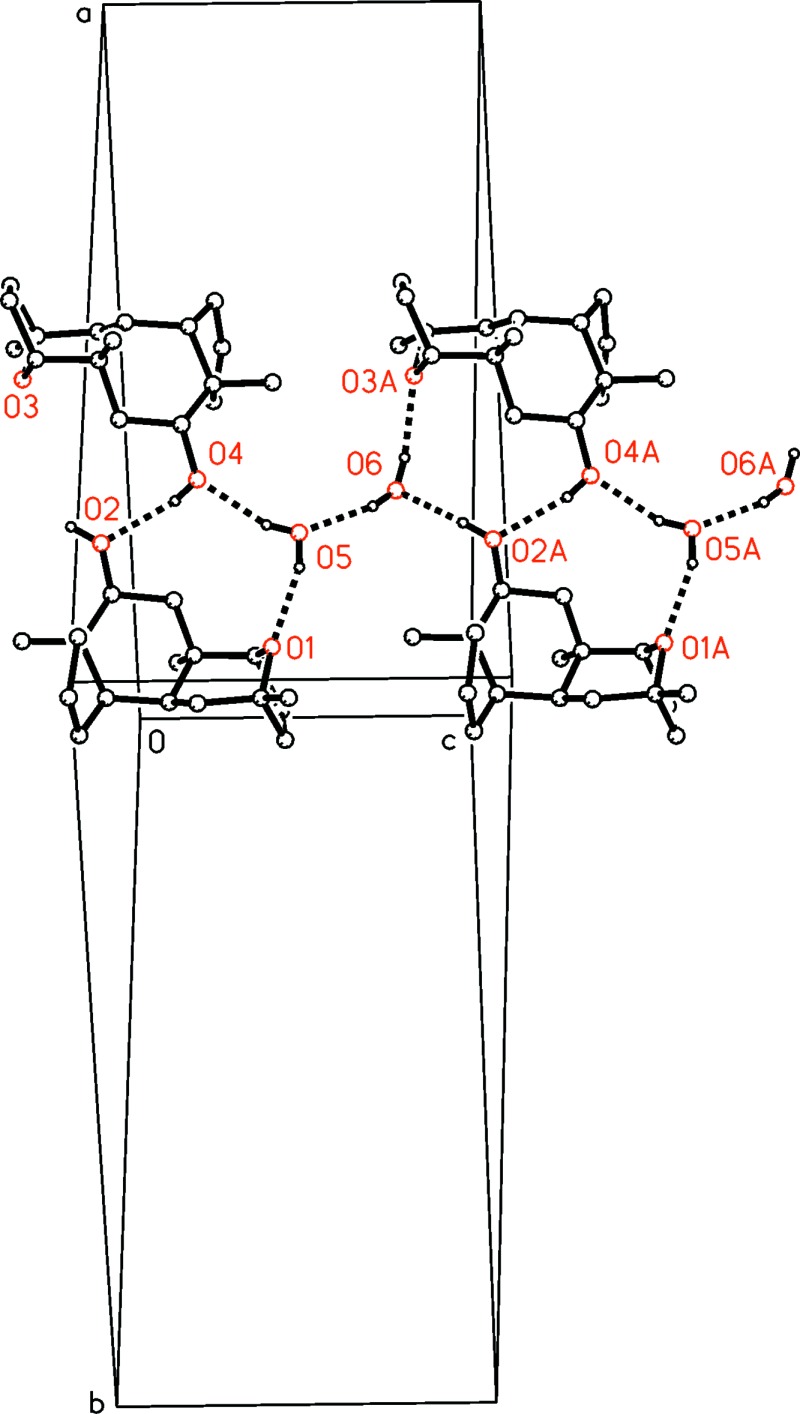
: Unit cell showing the 1-D network of molecules linked by intermolecular O—H ···O hydrogen bonds along crystallographic c axis. Hydrogen bonds between the C17H28O2 molecules, between C17H28O2 and water molecules and between water molecules are shown as dotted lines. H atoms not involved are omitted.

. DOI: 10.1107/S2056989015015698/fk2090fig1.tif
The mol­ecular structure of the title compound with anisotropic displacement ellipsoids drawn at the 50% probability level.

c 17 28 2 17 28 2 . DOI: 10.1107/S2056989015015698/fk2090fig2.tif
Unit cell showing the 1-D network of mol­ecules linked by inter­molecular O—H ⋯O hydrogen bonds along crystallographic *c* axis. Hydrogen bonds between the C_17_H_28_O_2_ mol­ecules, between C_17_H_28_O_2_ and water mol­ecules and between water mol­ecules are shown as dotted lines. H atoms not involved are omitted.

CCDC reference: 1419938

Additional supporting information:  crystallographic information; 3D view; checkCIF report

## Figures and Tables

**Table 1:** Hydrogen-bond geometry (, )

*D*H*A*	*D*H	H*A*	*D* *A*	*D*H*A*
O2H2O6^i^	0.85(1)	1.93(2)	2.770(4)	171(4)
O6H6*A*O3^ii^	0.84(1)	2.03(1)	2.874(3)	176(4)
O4H4O2	0.84(1)	2.03(2)	2.871(3)	176(4)
O5H5*A*O1	0.85(1)	2.06(2)	2.894(3)	169(5)
O6H6*B*O5	0.85(1)	1.99(2)	2.826(4)	172(5)
O5H5*B*O4	0.85(1)	1.96(2)	2.809(4)	178(5)

Symmetry codes: (i) 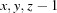; (ii) 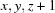.

## supplementary crystallographic information

### S1. Comment

As part of our studies in the total synthesis of diterpenoids, we afforded the 5,6,6,5-tetracyclic compound *rac*-(3a*R*,4*S*,5a*R*,6*S*,9*R*,10*aS*,10*bR*)-3a,5a,9-trimethyltetradecahydro-6,9-epoxycyclohepta[*e*]inden-4-yl 4-bromobenzoate (II). The saponification of (II) with sodium hydroxide in methanol provided *rac*-(3a*R*,4*S*,5a*R*,6*S*,9*R*,10aS,10*bR*)-3a,5a,9-trimethyltetradecahydro-6,9-epoxycyclohepta[*e*]inden-4-ol (I) as a white solid. Subsequent crystallization of (I) under air delivered the title hydrate.

### S2. Experimental

A sealable glass pressure tube was charged with freshly pestled sodium hydroxide (NaOH, *M* = 39.997 g/mol, 107 mg, 2.682 mmol, 50 eq) in dry methanol (6.8 ml, 125 ml/mmol). The suspension was stirred at room temperature until a clear colorless solution appeared (10 min). Then a solution of *rac*-(3a*R*,4*S*,5a*R*,6*S*,9*R*,\ 10*aS*,10*bR*)-3a,5a,9-trimethyl\ tetradecahydro-6,9-epoxycyclohepta[*e*]inden-4-yl 4-bromobenzoate (II) (C_24_H_31_BrO_3_, *M* = 447.41 g/mol, 24.0 mg, 53.6 µmol, 1 eq) in methanol (6.8 ml, 125 ml/mmol) was added at 273 K. The cooling bath was removed and stirring was continued for 2 h at room temperature. Next, the tube was sealed with a Teflon screw cap and placed in a pre-heated oil bath (333 K). After being stirred for 17 h at 333 K, the reaction mixture was cooled to ambient temperature and diluted with saturated aqueous NH_4_Cl solution, water and dichloromethane. The resulting mixture was vigorously stirred for 15 min at room temperature. The phases were separated and the aqueous layer was extracted with CH_2_Cl_2_ (3x). The combined organic phases were dried (MgSO_4_) and the product was loaded directly onto silica gel. After removal of the volatiles under reduced pressure, purification by flash chromatography (cyclohexane/ethyl acetate 10/1 to 1/1) delivered *rac*-(3a*R*,4*S*,5a*R*,6*S*,\ 9*R*,10*aS*,10*bR*)-3a,5a,9-trimethyltetradecahydro-\ 6,9-epoxycyclohepta[*e*]inden-4-ol (I) (C_17_H_28_O_2_, *M* = 264.41 g/mol, 11.0 mg, 41.6 µmol, 78%) as a white solid. Crystallization of (I) from diethyl ether (5 ml, 120 ml/mmol) by slow evaporation under air provided colourless plates of the title hydrate. *R*_f_ 0.29 (cyclohexane/ethyl acetate 2/1); m.p. (monohydrate) 390–392 K; ^1^H NMR (CDCl_3_, 500 MHz) δ 0.83 (s, 3H), 1.07 (s, 3H), 1.22 (ddd, *J* = 13.0 Hz, *J* = 5.3 Hz, *J* = 1.1 Hz, 1H), 1.30–1.34 (m, 2H), 1.31 (s, 3H), 1.42–1.76 (m, 9H), 1.80–1.90 (m, 2H), 1.92–1.99 (m, 2H), 2.13 (dd, *J* = 13.0 Hz, *J* = 12.4 Hz, 1H), 3.64 (dd, *J* = 6.4 Hz, *J* = 1.6 Hz, 1H), 3.85 (dd, *J* = 12.4 Hz, *J* = 5.3 Hz, 1H); ^13^C NMR (CDCl_3_, 126 MHz) δ 20.9 (CH_3_), 21.1 (CH_2_), 26.1 (CH_2_), 26.9 (CH_2_), 27.0 (CH_3_), 28.2 (CH_3_), 31.6 (CH_2_), 34.2 (CH_2_), 35.0 (CH_2_), 36.9 (CH_2_), 37.3 (CH), 38.9 (C), 45.7 (CH), 48.2 (C), 74.9 (CH), 80.4 (C), 84.5 (CH); IR ν 3435 (*s*), 2955 (*s*), 2925 (*s*), 2870 (*s*), 1470 (*s*), 1450 (*s*), 1375 (*s*), 1235 (*m*), 1165 (*m*), 1085 (*s*), 1030 (*s*), 1010 (*s*), 810 (*m*), 730 (*m*); HRMS (ESI) Calcd. for C_17_H_29_O_2_ ([*M*+H]^+^): 265.21621; Found: 265.21672; Anal. Calcd. for C_17_H_30_O_3_ (monohydrate): C, 72.3; H, 10.7; Found: C, 72.1; H, 10.4.

### S3. Refinement

H-atoms attached to C, except those of CH_3_, were placed at calculated positions (C—H = 0.99 - 1.00 Å and *U*_iso_(H) = 1.2 *U*_eq_(C)), while those attached to O were taken from a difference map and isotropically refined with constraints (*DFIX* 0.84 0.01 H O). All CH_3_ hydrogen atoms, which were taken from a Fourier map (AFIX 137), were allowed to rotate but not to tip (C—H = 0.98 Å and *U*_iso_(H) = 1.5 *U*_eq_(C)). The structure could be solved in the space groups I-4 and I4_1_/a. The refinement in the acentric space group gave significantly better results and these are used in this paper. Refinement in I4_1_/a was unstable, gave ADPs with several cigar-shaped ellipsoids and worse values for R1, wR2 and S. As in the absence of significant anomalous scattering effects the Flack parameter is essentially meaningless, Friedel pairs were merged.

### Crystal data

**Table:**

C_17_H_28_O_2_·H_2_O	*D*_x_ = 1.209 Mg m^−^^3^
*M**_r_* = 282.41	Mo *K*α radiation, λ = 0.71073 Å
Tetragonal, *I*4	Cell parameters from 5041 reflections
*a* = 25.3756 (9) Å	θ = 2.5–25.8°
*c* = 9.6397 (6) Å	µ = 0.08 mm^−^^1^
*V* = 6207.2 (6) Å^3^	*T* = 173 K
*Z* = 16	Plank, colourless
*F*(000) = 2496	0.32 × 0.13 × 0.10 mm

### Data collection

**Table:**

Agilent Xcalibur Sapphire3 diffractometer	6781 independent reflections
Radiation source: Enhance (Mo) X-ray Source	4846 reflections with *I* > 2σ(*I*)
Graphite monochromator	*R*_int_ = 0.087
Detector resolution: 16.0560 pixels mm^-1^	θ_max_ = 27.0°, θ_min_ = 2.3°
ω scans	*h* = −31→31
Absorption correction: multi-scan (*CrysAlis PRO*; Agilent, 2014)	*k* = −32→32
*T*_min_ = 0.978, *T*_max_ = 1.0	*l* = −12→12
36469 measured reflections	

### Refinement

**Table:**

Refinement on *F*^2^	Primary atom site location: structure-invariant direct methods
Least-squares matrix: full	Secondary atom site location: difference Fourier map
*R*[*F*^2^ > 2σ(*F*^2^)] = 0.051	Hydrogen site location: mixed
*wR*(*F*^2^) = 0.109	H atoms treated by a mixture of independent and constrained refinement
*S* = 1.03	*w* = 1/[σ^2^(*F*_o_^2^) + (0.0458*P*)^2^] where *P* = (*F*_o_^2^ + 2*F*_c_^2^)/3
6781 reflections	(Δ/σ)_max_ < 0.001
391 parameters	Δρ_max_ = 0.19 e Å^−^^3^
6 restraints	Δρ_min_ = −0.27 e Å^−^^3^

### Special details

**Table:**

Experimental. Absorption correction: *CrysAlis PRO*, Agilent Technologies, Version 1.171.37.34 (release 22–05-2014 CrysAlis171. NET) (compiled May 22 2014,16:03:01) Empirical absorption correction using spherical harmonics, implemented in SCALE3 ABSPACK scaling algorithm.
Geometry. All e.s.d.'s (except the e.s.d. in the dihedral angle between two l.s. planes) are estimated using the full covariance matrix. The cell e.s.d.'s are taken into account individually in the estimation of e.s.d.'s in distances, angles and torsion angles; correlations between e.s.d.'s in cell parameters are only used when they are defined by crystal symmetry. An approximate (isotropic) treatment of cell e.s.d.'s is used for estimating e.s.d.'s involving l.s. planes.

### Fractional atomic coordinates and isotropic or equivalent isotropic displacement parameters (Å^2^)

**Table:**

	*x*	*y*	*z*	*U*_iso_*/*U*_eq_	
O1	0.66099 (9)	0.59249 (8)	0.4355 (3)	0.0213 (6)	
O2	0.73096 (9)	0.51802 (10)	0.0043 (3)	0.0258 (6)	
H2	0.7353 (16)	0.5049 (14)	−0.076 (2)	0.050 (13)*	
O6	0.75052 (11)	0.46581 (11)	0.7572 (3)	0.0304 (6)	
H6A	0.7767 (9)	0.4458 (11)	0.768 (4)	0.024 (10)*	
H6B	0.7522 (18)	0.4812 (16)	0.679 (3)	0.061 (16)*	
C1	0.67949 (15)	0.68384 (14)	0.4781 (4)	0.0300 (9)	
H1A	0.7150	0.6801	0.4391	0.045*	
H1B	0.6804	0.6761	0.5777	0.045*	
H1C	0.6670	0.7200	0.4637	0.045*	
C2	0.64250 (13)	0.64565 (13)	0.4066 (4)	0.0216 (8)	
C3	0.58635 (13)	0.64494 (13)	0.4660 (4)	0.0263 (8)	
H3A	0.5625	0.6675	0.4105	0.032*	
H3B	0.5860	0.6572	0.5635	0.032*	
C4	0.56997 (13)	0.58639 (13)	0.4558 (4)	0.0268 (8)	
H4A	0.5624	0.5716	0.5488	0.032*	
H4B	0.5384	0.5821	0.3964	0.032*	
C5	0.61801 (13)	0.55991 (13)	0.3907 (4)	0.0213 (8)	
H5	0.6222	0.5237	0.4299	0.026*	
C6	0.61716 (12)	0.55698 (12)	0.2318 (4)	0.0177 (7)	
C7	0.57268 (13)	0.51931 (13)	0.1867 (4)	0.0280 (9)	
H7A	0.5720	0.5170	0.0853	0.042*	
H7B	0.5388	0.5328	0.2202	0.042*	
H7C	0.5790	0.4843	0.2260	0.042*	
C8	0.60877 (12)	0.61283 (12)	0.1714 (4)	0.0183 (8)	
H8	0.5714	0.6225	0.1923	0.022*	
C9	0.64334 (12)	0.65322 (13)	0.2491 (4)	0.0191 (8)	
H9A	0.6801	0.6501	0.2157	0.023*	
H9B	0.6308	0.6892	0.2269	0.023*	
C10	0.61376 (12)	0.61247 (12)	0.0109 (4)	0.0173 (8)	
H10	0.5813	0.5957	−0.0278	0.021*	
C11	0.61827 (13)	0.66842 (13)	−0.0518 (4)	0.0232 (8)	
H11A	0.6002	0.6702	−0.1427	0.028*	
H11B	0.6023	0.6948	0.0110	0.028*	
C12	0.67794 (14)	0.67872 (13)	−0.0688 (4)	0.0263 (9)	
H12A	0.6855	0.6929	−0.1624	0.032*	
H12B	0.6902	0.7046	0.0011	0.032*	
C13	0.70590 (13)	0.62545 (12)	−0.0479 (4)	0.0232 (8)	
H13A	0.7247	0.6248	0.0420	0.028*	
H13B	0.7317	0.6191	−0.1232	0.028*	
C14	0.66210 (13)	0.58337 (13)	−0.0508 (4)	0.0191 (8)	
C15	0.67692 (12)	0.53245 (13)	0.0275 (4)	0.0190 (8)	
H15	0.6540	0.5032	−0.0071	0.023*	
C16	0.67084 (13)	0.53596 (13)	0.1838 (4)	0.0193 (8)	
H16A	0.6988	0.5592	0.2208	0.023*	
H16B	0.6762	0.5005	0.2239	0.023*	
C17	0.65058 (14)	0.56877 (15)	−0.2027 (4)	0.0284 (9)	
H17A	0.6438	0.6009	−0.2562	0.043*	
H17B	0.6196	0.5458	−0.2067	0.043*	
H17C	0.6810	0.5503	−0.2420	0.043*	
O3	0.84108 (9)	0.40046 (9)	−0.1931 (3)	0.0228 (6)	
O4	0.78753 (10)	0.48936 (10)	0.2508 (3)	0.0308 (6)	
H4	0.7724 (16)	0.4988 (17)	0.178 (3)	0.062 (16)*	
O5	0.74801 (11)	0.52401 (11)	0.5073 (3)	0.0349 (7)	
H5A	0.7219 (12)	0.5445 (15)	0.499 (6)	0.084 (19)*	
H5B	0.7591 (19)	0.5138 (19)	0.429 (3)	0.088 (19)*	
C18	0.80880 (15)	0.31274 (14)	−0.2298 (5)	0.0339 (10)	
H18A	0.8093	0.3188	−0.3302	0.051*	
H18B	0.7745	0.3232	−0.1923	0.051*	
H18C	0.8149	0.2753	−0.2108	0.051*	
C19	0.85175 (13)	0.34518 (13)	−0.1618 (4)	0.0224 (8)	
C20	0.90685 (14)	0.33742 (14)	−0.2224 (4)	0.0301 (9)	
H20A	0.9049	0.3251	−0.3197	0.036*	
H20B	0.9271	0.3115	−0.1674	0.036*	
C21	0.93216 (14)	0.39215 (14)	−0.2139 (4)	0.0298 (9)	
H21A	0.9423	0.4050	−0.3072	0.036*	
H21B	0.9638	0.3916	−0.1537	0.036*	
C22	0.88853 (13)	0.42672 (14)	−0.1508 (4)	0.0244 (8)	
H22	0.8899	0.4628	−0.1924	0.029*	
C23	0.89047 (13)	0.43070 (13)	0.0088 (4)	0.0209 (8)	
C24	0.93975 (13)	0.46248 (14)	0.0502 (4)	0.0314 (9)	
H24A	0.9382	0.4975	0.0075	0.047*	
H24B	0.9714	0.4440	0.0182	0.047*	
H24C	0.9410	0.4662	0.1514	0.047*	
C25	0.89094 (12)	0.37507 (13)	0.0714 (4)	0.0195 (8)	
H25	0.9265	0.3601	0.0502	0.023*	
C26	0.85069 (13)	0.33920 (13)	−0.0037 (4)	0.0215 (8)	
H26A	0.8582	0.3020	0.0204	0.026*	
H26B	0.8149	0.3476	0.0303	0.026*	
C27	0.88639 (12)	0.37606 (12)	0.2315 (4)	0.0194 (8)	
H27	0.9210	0.3880	0.2699	0.023*	
C28	0.87333 (13)	0.32212 (13)	0.2959 (4)	0.0251 (8)	
H28A	0.8855	0.2933	0.2347	0.030*	
H28B	0.8906	0.3183	0.3874	0.030*	
C29	0.81308 (14)	0.32045 (13)	0.3117 (4)	0.0272 (9)	
H29A	0.8033	0.3077	0.4053	0.033*	
H29B	0.7974	0.2965	0.2418	0.033*	
C30	0.79305 (13)	0.37761 (13)	0.2896 (4)	0.0237 (8)	
H30A	0.7747	0.3809	0.1994	0.028*	
H30B	0.7684	0.3878	0.3645	0.028*	
C31	0.84251 (12)	0.41241 (13)	0.2926 (4)	0.0200 (8)	
C32	0.83662 (13)	0.46477 (13)	0.2139 (4)	0.0236 (8)	
H32	0.8657	0.4886	0.2451	0.028*	
C33	0.84067 (13)	0.45958 (13)	0.0579 (4)	0.0207 (8)	
H33A	0.8402	0.4953	0.0163	0.025*	
H33B	0.8093	0.4404	0.0234	0.025*	
C34	0.85574 (14)	0.42541 (14)	0.4452 (4)	0.0296 (9)	
H34A	0.8560	0.3929	0.4999	0.044*	
H34B	0.8291	0.4495	0.4825	0.044*	
H34C	0.8905	0.4421	0.4500	0.044*	

### Atomic displacement parameters (Å^2^)

**Table:**

	*U*^11^	*U*^22^	*U*^33^	*U*^12^	*U*^13^	*U*^23^
O1	0.0250 (13)	0.0208 (12)	0.0182 (14)	0.0045 (10)	−0.0002 (10)	0.0000 (10)
O2	0.0251 (14)	0.0325 (15)	0.0199 (15)	0.0134 (11)	0.0007 (11)	−0.0012 (12)
O6	0.0295 (15)	0.0372 (16)	0.0245 (16)	0.0122 (13)	0.0004 (13)	−0.0016 (13)
C1	0.039 (2)	0.032 (2)	0.019 (2)	0.0025 (17)	−0.0010 (17)	−0.0052 (17)
C2	0.0256 (19)	0.0194 (18)	0.020 (2)	0.0064 (15)	0.0000 (15)	−0.0027 (15)
C3	0.029 (2)	0.030 (2)	0.020 (2)	0.0094 (15)	0.0055 (16)	−0.0004 (17)
C4	0.0252 (19)	0.032 (2)	0.023 (2)	0.0007 (16)	0.0059 (16)	0.0034 (16)
C5	0.0217 (19)	0.0206 (19)	0.022 (2)	−0.0002 (15)	0.0013 (15)	0.0039 (15)
C6	0.0172 (17)	0.0171 (17)	0.0188 (19)	−0.0006 (13)	0.0027 (15)	0.0025 (14)
C7	0.030 (2)	0.026 (2)	0.028 (2)	−0.0068 (16)	0.0019 (17)	0.0016 (17)
C8	0.0112 (16)	0.0232 (18)	0.020 (2)	0.0025 (14)	0.0017 (14)	−0.0004 (15)
C9	0.0183 (17)	0.0175 (17)	0.022 (2)	0.0013 (14)	0.0008 (15)	0.0008 (15)
C10	0.0163 (17)	0.0214 (18)	0.0143 (19)	0.0006 (14)	−0.0020 (14)	0.0009 (14)
C11	0.0257 (19)	0.0263 (19)	0.018 (2)	0.0058 (15)	0.0009 (16)	0.0027 (15)
C12	0.031 (2)	0.0223 (19)	0.025 (2)	−0.0002 (15)	0.0020 (17)	0.0053 (16)
C13	0.0222 (19)	0.0278 (19)	0.020 (2)	0.0021 (15)	0.0042 (16)	0.0047 (17)
C14	0.0192 (18)	0.0222 (18)	0.0159 (19)	0.0056 (14)	0.0004 (15)	−0.0004 (15)
C15	0.0176 (17)	0.0201 (18)	0.0192 (19)	0.0042 (13)	0.0004 (14)	−0.0012 (15)
C16	0.0198 (18)	0.0198 (18)	0.0183 (19)	0.0017 (14)	−0.0012 (14)	0.0022 (15)
C17	0.030 (2)	0.036 (2)	0.020 (2)	0.0096 (16)	−0.0001 (17)	−0.0002 (17)
O3	0.0232 (13)	0.0230 (13)	0.0224 (14)	0.0051 (10)	−0.0009 (11)	0.0018 (11)
O4	0.0348 (15)	0.0374 (15)	0.0201 (15)	0.0189 (12)	−0.0001 (12)	−0.0016 (12)
O5	0.0362 (17)	0.0435 (17)	0.0249 (17)	0.0149 (14)	−0.0010 (14)	−0.0008 (14)
C18	0.039 (2)	0.032 (2)	0.031 (2)	0.0047 (18)	0.0008 (19)	−0.0083 (18)
C19	0.025 (2)	0.0228 (19)	0.019 (2)	0.0065 (15)	0.0024 (15)	0.0023 (16)
C20	0.032 (2)	0.035 (2)	0.023 (2)	0.0110 (17)	0.0044 (17)	0.0010 (18)
C21	0.025 (2)	0.045 (2)	0.020 (2)	0.0035 (17)	0.0083 (15)	0.0069 (18)
C22	0.0221 (19)	0.026 (2)	0.025 (2)	0.0011 (16)	0.0050 (16)	0.0076 (16)
C23	0.0186 (18)	0.0193 (18)	0.025 (2)	−0.0003 (14)	0.0019 (15)	0.0054 (15)
C24	0.0239 (19)	0.032 (2)	0.038 (2)	−0.0071 (16)	0.0020 (18)	0.0081 (18)
C25	0.0149 (17)	0.0240 (19)	0.020 (2)	0.0041 (14)	0.0020 (15)	0.0051 (15)
C26	0.0237 (19)	0.0178 (18)	0.023 (2)	0.0006 (14)	0.0012 (16)	0.0012 (15)
C27	0.0157 (17)	0.0188 (18)	0.024 (2)	0.0023 (13)	−0.0029 (15)	0.0025 (15)
C28	0.029 (2)	0.0245 (19)	0.022 (2)	0.0068 (15)	0.0002 (17)	0.0048 (16)
C29	0.031 (2)	0.026 (2)	0.025 (2)	−0.0026 (16)	0.0046 (17)	0.0038 (17)
C30	0.0201 (18)	0.029 (2)	0.022 (2)	0.0010 (15)	0.0041 (16)	0.0026 (17)
C31	0.0197 (18)	0.0238 (19)	0.0167 (19)	0.0011 (14)	−0.0018 (15)	0.0003 (15)
C32	0.0193 (18)	0.0239 (19)	0.028 (2)	0.0027 (14)	−0.0030 (16)	−0.0018 (16)
C33	0.0236 (18)	0.0175 (17)	0.021 (2)	0.0032 (14)	−0.0020 (16)	0.0051 (15)
C34	0.033 (2)	0.031 (2)	0.025 (2)	0.0045 (17)	−0.0052 (18)	−0.0001 (18)

### Geometric parameters (Å, º)

**Table:**

O1—C5	1.435 (4)	O3—C22	1.435 (4)
O1—C2	1.455 (4)	O3—C19	1.460 (4)
O2—C15	1.437 (4)	O4—C32	1.438 (4)
O2—H2	0.846 (14)	O4—H4	0.838 (14)
O6—H6A	0.844 (13)	O5—H5A	0.845 (14)
O6—H6B	0.847 (14)	O5—H5B	0.848 (14)
C1—C2	1.515 (5)	C18—C19	1.515 (5)
C1—H1A	0.9800	C18—H18A	0.9800
C1—H1B	0.9800	C18—H18B	0.9800
C1—H1C	0.9800	C18—H18C	0.9800
C2—C9	1.530 (5)	C19—C20	1.528 (5)
C2—C3	1.536 (5)	C19—C26	1.532 (5)
C3—C4	1.546 (5)	C20—C21	1.532 (5)
C3—H3A	0.9900	C20—H20A	0.9900
C3—H3B	0.9900	C20—H20B	0.9900
C4—C5	1.527 (5)	C21—C22	1.538 (5)
C4—H4A	0.9900	C21—H21A	0.9900
C4—H4B	0.9900	C21—H21B	0.9900
C5—C6	1.533 (5)	C22—C23	1.542 (5)
C5—H5	1.0000	C22—H22	1.0000
C6—C16	1.535 (4)	C23—C25	1.535 (4)
C6—C7	1.542 (4)	C23—C33	1.536 (5)
C6—C8	1.547 (4)	C23—C24	1.541 (5)
C7—H7A	0.9800	C24—H24A	0.9800
C7—H7B	0.9800	C24—H24B	0.9800
C7—H7C	0.9800	C24—H24C	0.9800
C8—C9	1.543 (5)	C25—C26	1.548 (5)
C8—C10	1.553 (5)	C25—C27	1.548 (5)
C8—H8	1.0000	C25—H25	1.0000
C9—H9A	0.9900	C26—H26A	0.9900
C9—H9B	0.9900	C26—H26B	0.9900
C10—C11	1.547 (4)	C27—C28	1.539 (4)
C10—C14	1.550 (4)	C27—C31	1.561 (5)
C10—H10	1.0000	C27—H27	1.0000
C11—C12	1.545 (5)	C28—C29	1.537 (5)
C11—H11A	0.9900	C28—H28A	0.9900
C11—H11B	0.9900	C28—H28B	0.9900
C12—C13	1.540 (4)	C29—C30	1.551 (5)
C12—H12A	0.9900	C29—H29A	0.9900
C12—H12B	0.9900	C29—H29B	0.9900
C13—C14	1.542 (4)	C30—C31	1.535 (4)
C13—H13A	0.9900	C30—H30A	0.9900
C13—H13B	0.9900	C30—H30B	0.9900
C14—C17	1.539 (5)	C31—C32	1.537 (5)
C14—C15	1.543 (4)	C31—C34	1.545 (5)
C15—C16	1.517 (5)	C32—C33	1.513 (5)
C15—H15	1.0000	C32—H32	1.0000
C16—H16A	0.9900	C33—H33A	0.9900
C16—H16B	0.9900	C33—H33B	0.9900
C17—H17A	0.9800	C34—H34A	0.9800
C17—H17B	0.9800	C34—H34B	0.9800
C17—H17C	0.9800	C34—H34C	0.9800
			
C5—O1—C2	103.4 (2)	C22—O3—C19	103.4 (2)
C15—O2—H2	112 (3)	C32—O4—H4	108 (3)
H6A—O6—H6B	111 (4)	H5A—O5—H5B	111 (5)
C2—C1—H1A	109.5	C19—C18—H18A	109.5
C2—C1—H1B	109.5	C19—C18—H18B	109.5
H1A—C1—H1B	109.5	H18A—C18—H18B	109.5
C2—C1—H1C	109.5	C19—C18—H18C	109.5
H1A—C1—H1C	109.5	H18A—C18—H18C	109.5
H1B—C1—H1C	109.5	H18B—C18—H18C	109.5
O1—C2—C1	107.8 (3)	O3—C19—C18	107.4 (3)
O1—C2—C9	107.6 (3)	O3—C19—C20	102.4 (3)
C1—C2—C9	111.3 (3)	C18—C19—C20	115.0 (3)
O1—C2—C3	102.5 (3)	O3—C19—C26	107.3 (3)
C1—C2—C3	114.4 (3)	C18—C19—C26	111.3 (3)
C9—C2—C3	112.6 (3)	C20—C19—C26	112.6 (3)
C2—C3—C4	103.7 (3)	C19—C20—C21	104.3 (3)
C2—C3—H3A	111.0	C19—C20—H20A	110.9
C4—C3—H3A	111.0	C21—C20—H20A	110.9
C2—C3—H3B	111.0	C19—C20—H20B	110.9
C4—C3—H3B	111.0	C21—C20—H20B	110.9
H3A—C3—H3B	109.0	H20A—C20—H20B	108.9
C5—C4—C3	103.6 (3)	C20—C21—C22	103.7 (3)
C5—C4—H4A	111.0	C20—C21—H21A	111.0
C3—C4—H4A	111.0	C22—C21—H21A	111.0
C5—C4—H4B	111.0	C20—C21—H21B	111.0
C3—C4—H4B	111.0	C22—C21—H21B	111.0
H4A—C4—H4B	109.0	H21A—C21—H21B	109.0
O1—C5—C4	103.3 (3)	O3—C22—C21	103.1 (3)
O1—C5—C6	109.8 (3)	O3—C22—C23	109.9 (3)
C4—C5—C6	114.9 (3)	C21—C22—C23	114.2 (3)
O1—C5—H5	109.5	O3—C22—H22	109.8
C4—C5—H5	109.5	C21—C22—H22	109.8
C6—C5—H5	109.5	C23—C22—H22	109.8
C5—C6—C16	107.8 (3)	C25—C23—C33	108.9 (3)
C5—C6—C7	108.8 (3)	C25—C23—C24	111.9 (3)
C16—C6—C7	110.4 (3)	C33—C23—C24	109.8 (3)
C5—C6—C8	109.5 (3)	C25—C23—C22	109.4 (3)
C16—C6—C8	109.1 (3)	C33—C23—C22	108.2 (3)
C7—C6—C8	111.2 (3)	C24—C23—C22	108.6 (3)
C6—C7—H7A	109.5	C23—C24—H24A	109.5
C6—C7—H7B	109.5	C23—C24—H24B	109.5
H7A—C7—H7B	109.5	H24A—C24—H24B	109.5
C6—C7—H7C	109.5	C23—C24—H24C	109.5
H7A—C7—H7C	109.5	H24A—C24—H24C	109.5
H7B—C7—H7C	109.5	H24B—C24—H24C	109.5
C9—C8—C6	110.3 (3)	C23—C25—C26	110.6 (3)
C9—C8—C10	116.2 (3)	C23—C25—C27	112.1 (3)
C6—C8—C10	111.0 (3)	C26—C25—C27	115.2 (3)
C9—C8—H8	106.2	C23—C25—H25	106.1
C6—C8—H8	106.2	C26—C25—H25	106.1
C10—C8—H8	106.2	C27—C25—H25	106.1
C2—C9—C8	112.9 (3)	C19—C26—C25	113.3 (3)
C2—C9—H9A	109.0	C19—C26—H26A	108.9
C8—C9—H9A	109.0	C25—C26—H26A	108.9
C2—C9—H9B	109.0	C19—C26—H26B	108.9
C8—C9—H9B	109.0	C25—C26—H26B	108.9
H9A—C9—H9B	107.8	H26A—C26—H26B	107.7
C11—C10—C14	103.2 (3)	C28—C27—C25	113.8 (3)
C11—C10—C8	112.9 (3)	C28—C27—C31	102.7 (3)
C14—C10—C8	116.7 (3)	C25—C27—C31	116.0 (3)
C11—C10—H10	107.9	C28—C27—H27	108.0
C14—C10—H10	107.9	C25—C27—H27	108.0
C8—C10—H10	107.9	C31—C27—H27	108.0
C12—C11—C10	105.6 (3)	C29—C28—C27	106.2 (3)
C12—C11—H11A	110.6	C29—C28—H28A	110.5
C10—C11—H11A	110.6	C27—C28—H28A	110.5
C12—C11—H11B	110.6	C29—C28—H28B	110.5
C10—C11—H11B	110.6	C27—C28—H28B	110.5
H11A—C11—H11B	108.7	H28A—C28—H28B	108.7
C13—C12—C11	106.8 (3)	C28—C29—C30	106.6 (3)
C13—C12—H12A	110.4	C28—C29—H29A	110.4
C11—C12—H12A	110.4	C30—C29—H29A	110.4
C13—C12—H12B	110.4	C28—C29—H29B	110.4
C11—C12—H12B	110.4	C30—C29—H29B	110.4
H12A—C12—H12B	108.6	H29A—C29—H29B	108.6
C12—C13—C14	105.9 (3)	C31—C30—C29	105.5 (3)
C12—C13—H13A	110.6	C31—C30—H30A	110.6
C14—C13—H13A	110.6	C29—C30—H30A	110.6
C12—C13—H13B	110.6	C31—C30—H30B	110.6
C14—C13—H13B	110.6	C29—C30—H30B	110.6
H13A—C13—H13B	108.7	H30A—C30—H30B	108.8
C17—C14—C13	108.7 (3)	C30—C31—C32	114.1 (3)
C17—C14—C15	108.1 (3)	C30—C31—C34	108.6 (3)
C13—C14—C15	113.3 (3)	C32—C31—C34	107.8 (3)
C17—C14—C10	109.2 (3)	C30—C31—C27	103.7 (3)
C13—C14—C10	103.5 (3)	C32—C31—C27	113.2 (3)
C15—C14—C10	113.9 (3)	C34—C31—C27	109.3 (3)
O2—C15—C16	105.5 (3)	O4—C32—C33	110.1 (3)
O2—C15—C14	111.7 (3)	O4—C32—C31	109.7 (3)
C16—C15—C14	114.3 (3)	C33—C32—C31	114.1 (3)
O2—C15—H15	108.4	O4—C32—H32	107.6
C16—C15—H15	108.4	C33—C32—H32	107.6
C14—C15—H15	108.4	C31—C32—H32	107.6
C15—C16—C6	114.2 (3)	C32—C33—C23	113.8 (3)
C15—C16—H16A	108.7	C32—C33—H33A	108.8
C6—C16—H16A	108.7	C23—C33—H33A	108.8
C15—C16—H16B	108.7	C32—C33—H33B	108.8
C6—C16—H16B	108.7	C23—C33—H33B	108.8
H16A—C16—H16B	107.6	H33A—C33—H33B	107.7
C14—C17—H17A	109.5	C31—C34—H34A	109.5
C14—C17—H17B	109.5	C31—C34—H34B	109.5
H17A—C17—H17B	109.5	H34A—C34—H34B	109.5
C14—C17—H17C	109.5	C31—C34—H34C	109.5
H17A—C17—H17C	109.5	H34A—C34—H34C	109.5
H17B—C17—H17C	109.5	H34B—C34—H34C	109.5
			
C5—O1—C2—C1	167.9 (3)	C22—O3—C19—C18	−168.4 (3)
C5—O1—C2—C9	−72.1 (3)	C22—O3—C19—C20	−46.9 (3)
C5—O1—C2—C3	46.8 (3)	C22—O3—C19—C26	71.8 (3)
O1—C2—C3—C4	−27.2 (3)	O3—C19—C20—C21	28.1 (4)
C1—C2—C3—C4	−143.6 (3)	C18—C19—C20—C21	144.3 (3)
C9—C2—C3—C4	88.1 (3)	C26—C19—C20—C21	−86.8 (3)
C2—C3—C4—C5	−0.5 (4)	C19—C20—C21—C22	−0.8 (4)
C2—O1—C5—C4	−47.5 (3)	C19—O3—C22—C21	46.5 (3)
C2—O1—C5—C6	75.5 (3)	C19—O3—C22—C23	−75.6 (3)
C3—C4—C5—O1	28.6 (3)	C20—C21—C22—O3	−27.3 (4)
C3—C4—C5—C6	−91.0 (3)	C20—C21—C22—C23	91.9 (4)
O1—C5—C6—C16	57.0 (3)	O3—C22—C23—C25	61.6 (3)
C4—C5—C6—C16	172.9 (3)	C21—C22—C23—C25	−53.6 (4)
O1—C5—C6—C7	176.8 (2)	O3—C22—C23—C33	−56.9 (4)
C4—C5—C6—C7	−67.3 (4)	C21—C22—C23—C33	−172.2 (3)
O1—C5—C6—C8	−61.5 (3)	O3—C22—C23—C24	−176.0 (2)
C4—C5—C6—C8	54.3 (4)	C21—C22—C23—C24	68.7 (4)
C5—C6—C8—C9	43.5 (4)	C33—C23—C25—C26	74.8 (3)
C16—C6—C8—C9	−74.2 (3)	C24—C23—C25—C26	−163.7 (3)
C7—C6—C8—C9	163.8 (3)	C22—C23—C25—C26	−43.3 (4)
C5—C6—C8—C10	173.8 (3)	C33—C23—C25—C27	−55.3 (3)
C16—C6—C8—C10	56.1 (3)	C24—C23—C25—C27	66.2 (4)
C7—C6—C8—C10	−66.0 (3)	C22—C23—C25—C27	−173.4 (3)
O1—C2—C9—C8	58.6 (3)	O3—C19—C26—C25	−58.1 (4)
C1—C2—C9—C8	176.5 (3)	C18—C19—C26—C25	−175.4 (3)
C3—C2—C9—C8	−53.6 (4)	C20—C19—C26—C25	53.8 (4)
C6—C8—C9—C2	−43.9 (4)	C23—C25—C26—C19	43.8 (4)
C10—C8—C9—C2	−171.4 (3)	C27—C25—C26—C19	172.2 (3)
C9—C8—C10—C11	−39.9 (4)	C23—C25—C27—C28	166.1 (3)
C6—C8—C10—C11	−167.0 (2)	C26—C25—C27—C28	38.4 (4)
C9—C8—C10—C14	79.5 (4)	C23—C25—C27—C31	47.2 (4)
C6—C8—C10—C14	−47.6 (4)	C26—C25—C27—C31	−80.5 (4)
C14—C10—C11—C12	−31.8 (3)	C25—C27—C28—C29	−93.9 (3)
C8—C10—C11—C12	95.1 (3)	C31—C27—C28—C29	32.3 (4)
C10—C11—C12—C13	12.5 (4)	C27—C28—C29—C30	−13.2 (4)
C11—C12—C13—C14	11.9 (4)	C28—C29—C30—C31	−11.7 (4)
C12—C13—C14—C17	84.5 (3)	C29—C30—C31—C32	155.2 (3)
C12—C13—C14—C15	−155.4 (3)	C29—C30—C31—C34	−84.5 (3)
C12—C13—C14—C10	−31.6 (3)	C29—C30—C31—C27	31.6 (3)
C11—C10—C14—C17	−76.7 (3)	C28—C27—C31—C30	−39.4 (3)
C8—C10—C14—C17	158.8 (3)	C25—C27—C31—C30	85.4 (3)
C11—C10—C14—C13	39.0 (3)	C28—C27—C31—C32	−163.6 (3)
C8—C10—C14—C13	−85.5 (3)	C25—C27—C31—C32	−38.8 (4)
C11—C10—C14—C15	162.5 (3)	C28—C27—C31—C34	76.2 (3)
C8—C10—C14—C15	38.0 (4)	C25—C27—C31—C34	−159.0 (3)
C17—C14—C15—O2	80.7 (3)	C30—C31—C32—O4	46.5 (4)
C13—C14—C15—O2	−39.8 (4)	C34—C31—C32—O4	−74.2 (3)
C10—C14—C15—O2	−157.8 (3)	C27—C31—C32—O4	164.7 (3)
C17—C14—C15—C16	−159.7 (3)	C30—C31—C32—C33	−77.5 (4)
C13—C14—C15—C16	79.8 (4)	C34—C31—C32—C33	161.8 (3)
C10—C14—C15—C16	−38.2 (4)	C27—C31—C32—C33	40.7 (4)
O2—C15—C16—C6	173.6 (2)	O4—C32—C33—C23	−176.6 (3)
C14—C15—C16—C6	50.5 (4)	C31—C32—C33—C23	−52.7 (4)
C5—C6—C16—C15	−178.1 (3)	C25—C23—C33—C32	59.4 (4)
C7—C6—C16—C15	63.2 (4)	C24—C23—C33—C32	−63.5 (4)
C8—C6—C16—C15	−59.3 (4)	C22—C23—C33—C32	178.2 (3)

### Hydrogen-bond geometry (Å, º)

**Table:**

*D*—H···*A*	*D*—H	H···*A*	*D*···*A*	*D*—H···*A*
O2—H2···O6^i^	0.85 (1)	1.93 (2)	2.770 (4)	171 (4)
O6—H6*A*···O3^ii^	0.84 (1)	2.03 (1)	2.874 (3)	176 (4)
O4—H4···O2	0.84 (1)	2.03 (2)	2.871 (3)	176 (4)
O5—H5*A*···O1	0.85 (1)	2.06 (2)	2.894 (3)	169 (5)
O6—H6*B*···O5	0.85 (1)	1.99 (2)	2.826 (4)	172 (5)
O5—H5*B*···O4	0.85 (1)	1.96 (2)	2.809 (4)	178 (5)

Symmetry codes: (i) *x*, *y*, *z*−1; (ii) *x*, *y*, *z*+1.
