# Supplementary figures and images for: Crystal structure of rac-(3aR,4S,5aR,6S,9R,10aS,10bR)-3a,5a,9-tri­methyl­tetra­deca­hydro-6,9-ep­oxy­cyclo­hepta­[e]inden-4-ol monohydrate
Source: Acta Crystallogr E Crystallogr Commun. 2015 Aug 29;71(Pt 9):o690–1. doi: 10.1107/S2056989015015698 (PMC4555390; doi:10.1107/S2056989015015698)

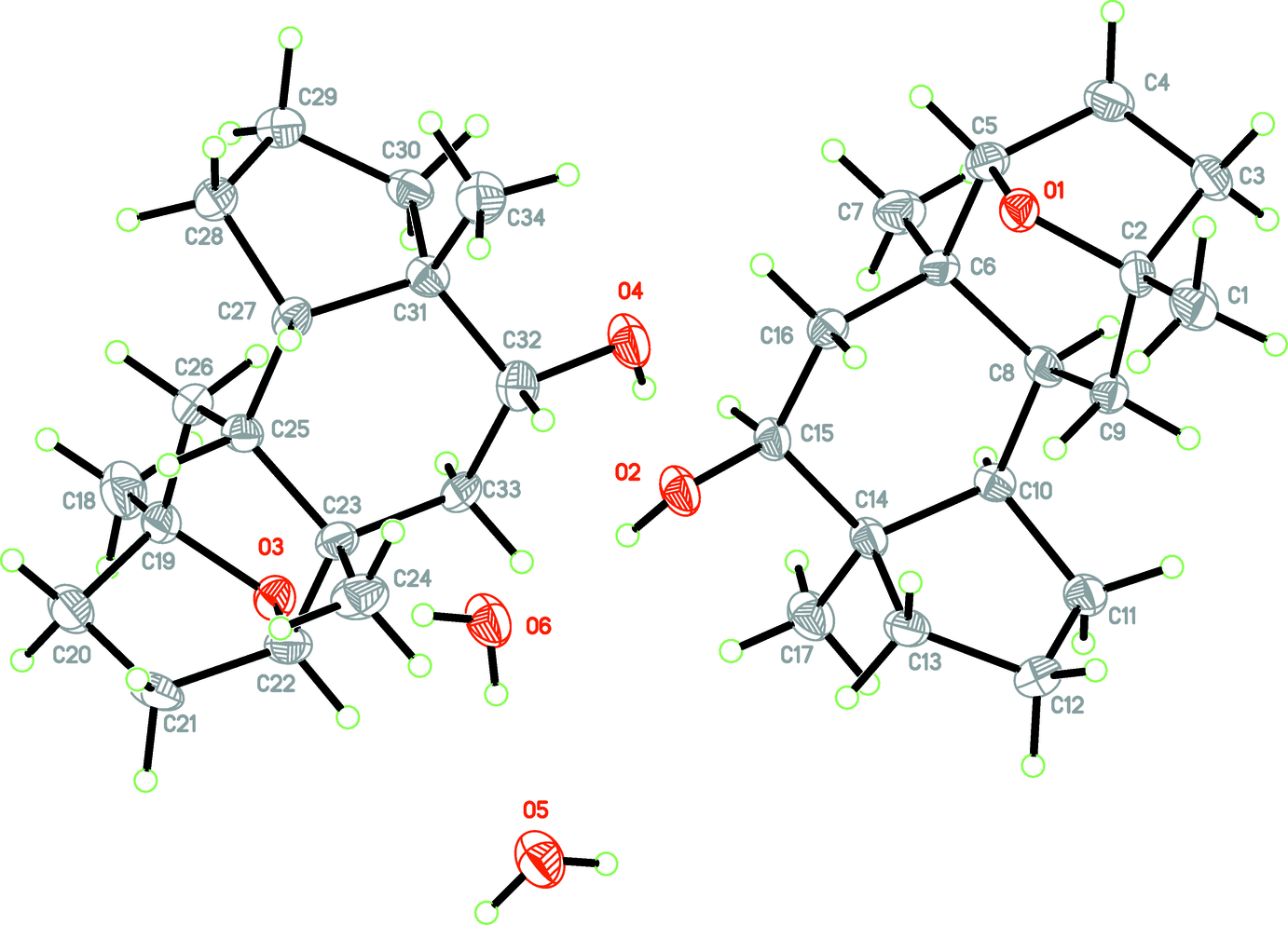

Supplement: Supplementary file 4 [file e-71-0o690-fig1.tif]

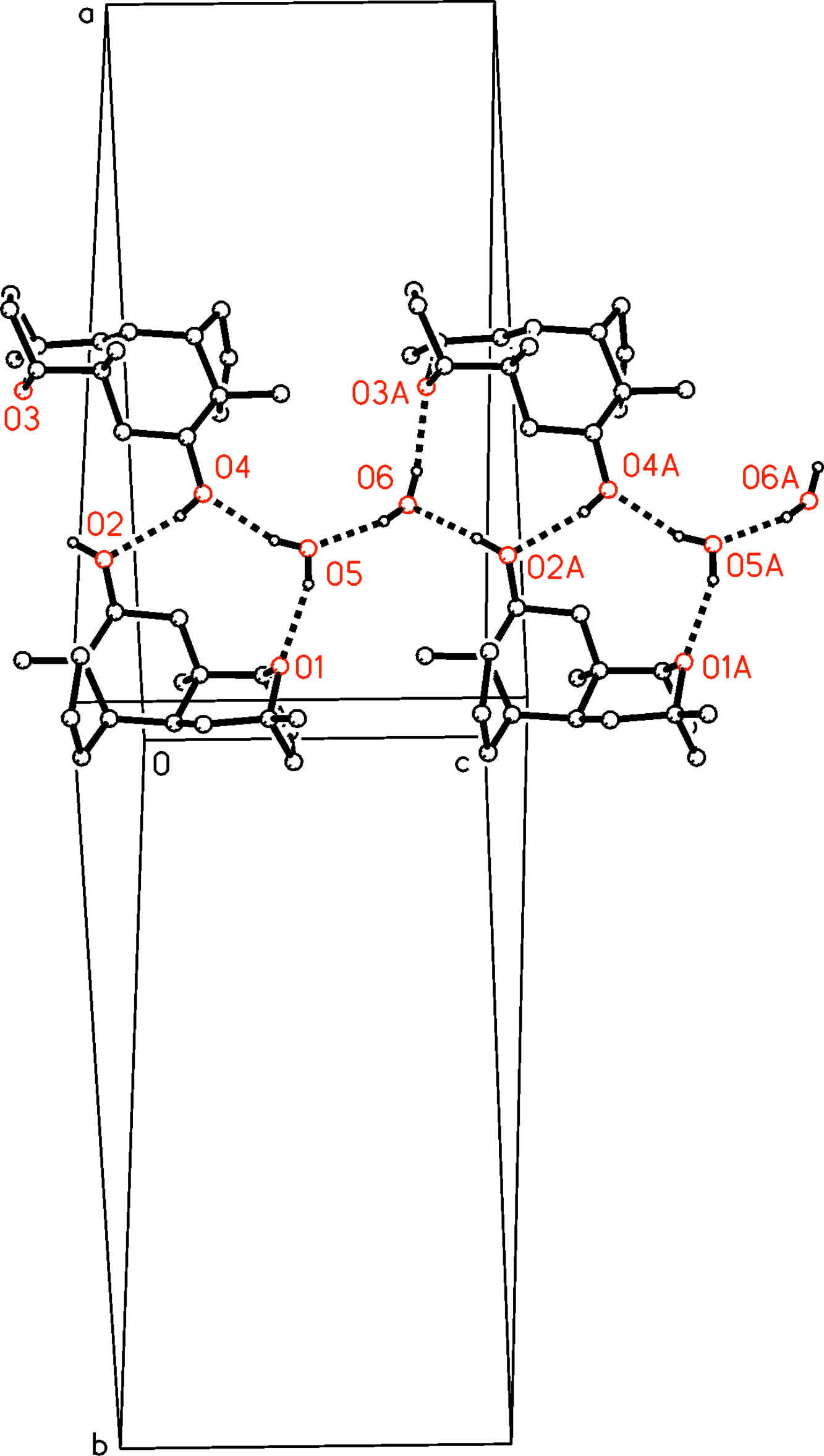

Supplement: Supplementary file 5 [file e-71-0o690-fig2.tif]
